# Bibliography

**Published:** 1911-03-15

**Authors:** 


### BIBLIOGRAPHY.

JOHNSONS OPERATIVE DENTISTRY. The second edition of this work follows the first edition so closely that there has been little chance to make extensive additions to its text, but the chapters on "Hygiene of the Mouth" and "Gold Inlays" have been revamped and brought up to date. The treatment however in both cases is entirely too brief to comport with the importance of these subjects. If we were to offer a criticism of this book, which is generally excellent, it would be that what we consider the most important subjects have received all too little consideration, whilst subjects that are already extensively treated in special works on their subjects are given the mo t extensive attention. For instance, the first eighty-three pages are devoted to anatomy and histology and the last two hundred and fifty pages are devoted to orthodontia. Thus giving up nearly one-half of its space to subjects not technically pertinent to a work on Operative Dentistry. Whilst the subject which one expects to find exhaustively treated; such as, the physical properties of various filling materials and the technical handling of these materials receive only the writer's methods, described so briefly, in some instances, as to seem elementary.

As a literary contribution it will add much to your resources but it can never in its present form become a valuable text book. The book is handsomely made, as the printing and illustrations are in most cases all that one could desire. Every practitioner should own this book as he will receive great benefit from reading the articles of some of the best workers in our profession on topics in which they are masters.

